# Head–Shaft Angle Influences Isometric Shoulder Strength Levels after Intramedullary Nailing of Proximal Humerus Fractures: A Pilot Study

**DOI:** 10.3390/jpm14090907

**Published:** 2024-08-27

**Authors:** Sebastian Grimme, Hermann Josef Bail, Johannes Rüther, Michael Millrose, Roland Biber, Markus Gesslein, Maximilian Willauschus

**Affiliations:** 1 Department of Orthopedics and Traumatology, Paracelsus Medical University, General Hospital Nuremberg, Breslauer Straße 201, 90471 Nuremberg, Germany; sebastian.grimme@usb.ch (S.G.); hermann-josef.bail@klinikum-nuernberg.de (H.J.B.); johannes.ruether@klinikum-nuernberg.de (J.R.); michael.millrose@klinikum-gap.de (M.M.); markus.gesslein@klinikum-nuernberg.de (M.G.); 2 Department of Orthopedics and Traumatology, University Hospital Basel, 4031 Basel, Switzerland; 3 Department of Trauma Surgery and Sports Medicine, Garmisch-Partenkirchen Medical Centre, 82467 Garmisch-Partenkirchen, Germany; 4 Department of Traumatology, Clinic Dr. Erler gGmbH, 90429 Nuremberg, Germany; r.biber@erler-klinik.de

**Keywords:** proximal humerus fracture, intramedullary nailing, head–shaft angle, head–neck angle, isometric strength, PROMs

## Abstract

Background: Proximal humerus fractures are common fractures of the elderly population which can lead to long-term compromise of a patient’s shoulder function. Closed reduction and internal fixation with intramedullary nailing is a well-established surgical technique yielding good outcomes, as perceived by patients, obtained via Patient-Reported Outcome Measures, and objectified by clinical shoulder testing. Apart from conventional range-of-motion testing and clinical shoulder tests, strength testing of the shoulder is a yet-neglected but meaningful and standardizable outcome parameter. In this study, isometric shoulder strength is evaluated in relation to fracture morphology/postoperative reduction quality as well as with patient-reported outcomes. Methods: 25 patients (mean age 73.2 ± 10.5 years) underwent isometrics strength-testing of the shoulder joint in the scapular plane (abduction) as well as in the sagittal plane (flexion) as well as hand-grip strength-testing at 4.5 ± 1.88 years follow-up. Pre- and postoperative radiographs were analysed. Patients completed ASES and CMS questionnaires. Results: Patients exhibited a decrease in abduction and flexion force (−24.47% and −25.30%, respectively, *p* < 0.001) using the contralateral, uninjured arm as reference. Abduction force tended to be decreased in three- and four-part fractures. Patient satisfaction correlated negatively with the relatively reduced force of the affected arm. Varus-angulated humeral heads produced significantly lower abduction force output than valgus- or physiologic angulation (*p* = 0.014), whereas flexion force was unaffected (*p* = 0.468). The anatomical reduction had no influence on shoulder strength. Conclusions: Proximal humerus fractures may cause a significant reduction in shoulder function, both reported by patients and objectified by shoulder strength testing. Varus head angulation demonstrated the greatest loss of shoulder strength and should be avoided to ensure proper functioning. Further, strength testing seems a valuable outcome parameter for a thorough shoulder examination with easy obtainability.

## 1. Introduction

Proximal humerus fractures (PHFs) are among the most common fractures affecting the elderly population [1,2,3]. Despite being an incisive event threatening one’s autonomy, the treatment of PHFs poses an increasing burden onto health-care expenditure and care availability. Against the background of a variety of surgical treatment options for displaced fractures of the surgical neck, intramedullary nailing (IMN) is a widely used technique yielding good to excellent outcomes for patients [4,5,6,7]. Hereby, a nail is inserted into the proximal medullary canal via the humeral head and fixated by interlocking screws to stabilize the repositioned fracture parts. A relatively small incision, soft tissue and nerve sparing, early postoperative physiotherapeutic exercise and a comparably short operation time represent the major advantages [8,9,10]. Several studies and meta-analyses have investigated outcomes based on subjective patient-centred or objective clinical measures. Established risk factors include insufficient anatomical reduction, four-part fractures, varus head–shaft angles (<130°) and the occurrence of complications [11,12]. Until now, strength measurements were commonly utilized for Constant–Murley score calculation, despite being an objective and easily measurable objective variable worthwhile addressing independently. While being linked with function, muscle strength is also a protective factor for falls, mobility, and independence [13,14]. Since it is reasonable that deficiencies in flexion and abduction force may impair activities of daily life, its magnitude and associations with other outcome parameters remain unclear. As previously described in the literature, isometric abduction force in the scapular plane and flexion force in the sagittal plane are well-established benchmarks for shoulder strength, the joint comprised by PHFs. Currently, there is limited knowledge regarding the factors that influence isometric strength following proximal humeral fractures. Additionally, there is a lack of published data concerning changes in shoulder strength after IMN.

Furthermore, since hand grip strength is a reliable indicator for frailty and adverse health outcomes, its assessment for potential declines following IMN gives valuable information concerning the long-term outcomes and compromises patients face [15].

The primary aim of this study was to evaluate the changes in shoulder strength and the according influence of anatomical reduction, fracture morphology and complications. The secondary aim was to investigate strength as related to Patient-Reported Outcome Measures (PROMs) following intramedullary nailing of proximal humerus fractures.

## 2. Materials and Methods

For this follow-up examination study, a total of 479 patients who underwent treatment with the Targon PH+ intramedullary nail (150 mm, Aesculap, B. Braun) for proximal humeral fractures (PHFs) at a single level-one trauma centre between 2014 and 2021 were considered. The study was approved by the institutional review committee (Paracelsus Medical University at the Nuremberg Hospital, Number IRB-2021-025).

Subsequently, exclusion criteria, as listed below, were applied, resulting in 260 patients who met the eligibility criteria for inclusion.

Exclusion criteria are comprised of patients unable to consent due to dementia and other neurologic diseases, concomitant shoulder injuries, insufficient clinical data or inadequate radiologic imaging, metastatic fractures, open fractures, additional shaft fractures, additional shoulder comorbidity (e.g., rotator cuff injuries, labral lesions), death in the follow-up period, conversion to shoulder prosthesis in the course of treatment, implantation of a nail length exceeding 150 mm and shoulder pathology at the contralateral arm. The inclusion criteria for this study encompassed patients with isolated proximal humeral fractures, unilateral injury, a minimum adequate radiological follow-up of more than one year, and provision of consent to participate. All patients who participated in the study provided written informed consent.

Thereafter, a cohort of 31 patients was successfully contacted and had provided informed consent to undergo a clinical examination and strength testing at our institution’s outpatient clinic. Two patients were excluded due to underlying conditions preventing load-bearing activities and four were excluded due to the implantation of a reverse shoulder prosthesis.

Patient’s contact information and general details were obtained from the clinic’s database and hospital information system, specifically by SAP (Wallendorf, Baden-Württemberg, Germany). Data analysis was performed using the statistical software IBM SPSS Statistics for Windows (version 28, 1.0.0.1406, IBM Corp., Armonk, NY, USA).

### 2.1. Surgical Technique

In every case, the decision for IMN was taken by a board of experienced orthopaedic and traumatological experts.

Prior to surgery, patients were positioned in beach chair position on a radiolucent table with a standard armrest. For implantation of the Targon PH+ nail, a deltoid split approach at the anterolateral margin of the acromion was performed. After a longitudinal transection of the clavipectoral fascia and the subacromial bursa, the head fragments were reduced via the joystick technique. For this procedure, a wire was used to adequately reduce the fragments.

After longitudinal splitting of the supraspinatus tendon, a guide pin at the apex of the humeral head was positioned after fluoroscopic control in two planes, and the nail was inserted 3 to 4 mm below the cartilage level. Then, a minimum of three to four locking head fixation screws were inserted into the head fragments, depending on the fracture pattern, the degree of instability, as well as bone quality.

A single or duplicate distal interlocking screw was placed through the nail percutaneously. Each procedure was performed by a board-certified trauma surgeon.

An intra- and postoperative x-ray examination was obtained from all patients to verify both the reduction and correct positioning of the implant.

The affected arm was immobilized in a sling bandage for one day to control postoperative pain and swelling. On the second postoperative day, passive mobilization by trained physiotherapists was started. An active-assisted shoulder movement below the pain threshold was then initiated as soon as wound healing was deemed satisfactory. After six weeks and radiological consolidation, weight-bearing was allowed. Every patient received postoperative standardized anterior-posterior and y-view radiographic imaging for correct fracture reposition and screw position. A radiographic follow-up after 12 months or longer was obtained from all patients.

### 2.2. Radiologic Fracture Assessment

**Figure 1:**
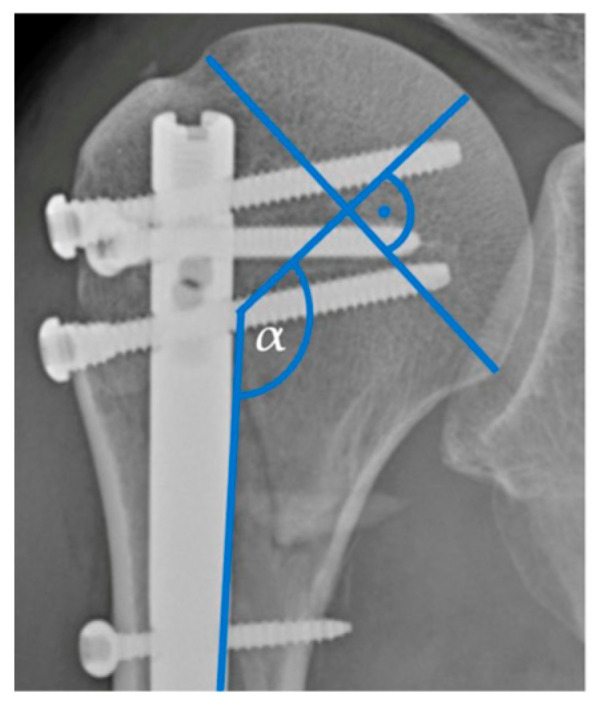
Head–shaft angle.

The pre- and postoperative radiographic imaging was assessed using Picture Archiving and Communication System software (Version: Ashvins 2017 Build 1637), which involved the analysis of plain radiographic images or computed tomography (CT) scans. Fractures were classified based on the number of fractured parts and according to the Neer classification system [16]. The analysis also focused on assessing the postoperative anatomical reposition of the fractures and the pre- and postoperative head–shaft angle (HSA) (Figure 1).

The follow-up radiographs were carefully evaluated by two experienced trauma surgeons to identify any documented complications. Anatomical reduction was considered when certain criteria had been met: the head showed neither varus angulation (<130°) nor valgus angulation (>140°), the y-view showed no anterior or posterior tilt of the head exceeding 20°, the dislocation of the greater or lesser tuberosity was less than 3 mm, and the neck–shaft dislocation was less than 5 mm. Early postoperative complications and revisions were evaluated during the hospital stay of the patients. These included reviews of postoperative X-rays and medical records. Later, complications and revisions were recorded from hospital charts during visits at our outpatient clinic or from readmission to any hospital and finally, at the point of follow-up. All complications were categorised according to the Clavien–Dindo classification and thereupon subdivided into major (Clavien–Dindo grade III and IV) and minor complications (Clavien–Dindo grade I and II) [17]. Every secondary surgery, besides elective implant removal, was counted as a revision. Implant failure was defined as the removal of the nail and/or conversion to a different osteosynthesis or the secondary implantation of a reverse shoulder prosthesis in the instance of unsatisfactory healing of the fracture site.

### 2.3. Examination of Patient-Related Outcome Measures

All patients signed the informed consent form and were reassured to feel capable of performing strength measurements. The validated German versions of the America Shoulder and Elbow Surgeons score (ASES) [18], the patient-reported part of Constant–Murley score (CMS), as well as patient’s satisfaction (very good, good, satisfactory, poor) were obtained. Other than ASES, besides Activities of Daily Life (ADL), CMS includes a physician-rated section including range of motion (ROM) (goniometer) and isometric strength measurements, thus being a “hybrid PROM”. After completing a baseline assessment for shoulder stability and ROM, a short warm-up routine was introduced.

The examination was performed with the patient in a standing position. Evaluation of pre-injury shoulder function was presumed by the function of the contralateral, i.e., unaffected arm.

**Figure 2:**
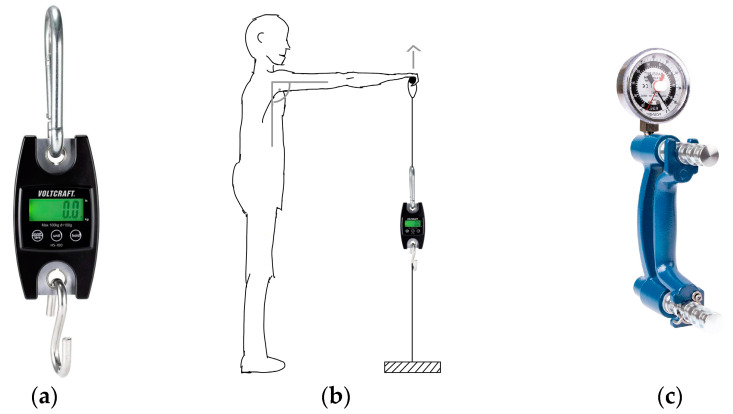
(**a**) VOLTCRAFT^®^ CE-certified electronic spring balance, with kind permission of Conrad Electronic SE; (**b**) Test set-up for flexion force measurement with the handheld handle attached to the spring balance; (**c**) BASELINE^®^ hand dynamometer, with kind permission of Sport-Tec GmbH (Pirmasens, Germany).

Bilateral isometric strength of both shoulder abduction in the scapular plane (kgs) and shoulder flexion (kgs) was assessed using the handheld CE-certified and calibrated electronic spring balance VOLTCRAFT HS-100 by Conrad^®^ (Hirschau, Bavaria, Germany) (see Figure 2a). To improve standardization, the handle length was adjusted according to the patient’s height. Beginning with the unaffected arm, patients were instructed to pull a handle with their straight arm parallel to the ground with maximum effort without provoking pain (see Figure 2b). Importantly, patients were instructed and observed not to augment their strength by contralateral torso inclination. Each, flexion force and abduction force were examined twice in an interval of one minute. The highest achieved orthogonal force was noted and interpreted as a measure of muscular strength (kgs).

### 2.4. Isometric Strength Measurements as a Proxy for Muscular Capability

Isometric strength measurements, although dependent on muscle length and position, show a high correlation with isokinetic measurements while being a more feasible option, since isokinetic measurement devices are rarely available in the clinical setting due to high acquisition costs. Both methods show clinical relevance and have been established for more than thirty years [19,20]. Further, isometric strength production has shown to be higher than isokinetic force production at any given joint’s angle [20].

Measuring isometric strength in the 90° abduction or flexion position after a proximal humeral fracture is crucial for assessing recovery, particularly focusing on the deltoid muscle, which is known to play a key role in shoulder abduction from 15° to 90°. At this angle, the deltoid’s lateral head is most active, making it an ideal position to evaluate its strength, especially considering the muscle’s significance following a fracture.

This position also offers joint stability, with the head of the humerus securely positioned within the glenoid fossa. This reduces the risk of subluxation or dislocation during the assessment. Furthermore, since daily activities often require arm movement at or above shoulder height, measuring strength at 90° provides valuable insights into functional recovery.

By choosing the 90° abduction or flexion position for measuring isometric strength, we also ensure that the assessment includes the scapulothoracic joint’s contribution to shoulder movement. In this position, the coordinated action of the trapezius and serratus anterior muscle is necessary to stabilize the scapula and allow smooth arm elevation. Assessing strength in this position helps evaluate not only the deltoid’s function but also the integrity of the scapulothoracic joint and the effectiveness of its muscle coordination, which are critical for overall shoulder function and stability post-fracture.

Bilateral hand grip strength was assessed with the calibrated BASELINE^®^ hydraulic hand dynamometer. After adjusting the handle grip width to hand size, subjects were instructed to compress the handle in a smooth motion in a 90° flexed elbow, whereby the measures were repeated three times to select the maximal score (kgs) (see Figure 2c).

### 2.5. Statistical Analysis

Because of not only sex-related but inter-individual strength differences of all subjects, it is requisite to introduce relative strength variables. Relative abduction force (AF_rel_), relative flexion force (FF_rel_) and relative hand grip force (HG_rel_) depict a quotient of the affected arm’s absolute force and the unaffected arm’s absolute force, resulting in a percentage allowing for comparability and statistical analysis. Further, relative strength values of a patient compared to their healthy, also presumably pre-injury arm, naturally show a higher clinical relevance than absolute values.

$$AFrel(\%)=\frac{Absolute\;flexion\;force\left(kgs\right)\;of\;the\;affected\;arm}{Absolute\;flexion\;force\left(kgs\right)\;of\;the\;unaffected\;arm}$$

Statistical analysis was performed using IMB SPSS Statistics for Mac (Version 29.0.2.0, IBM Corp., Armonk, NY, USA). All variables were tested for normal distribution. For force differences between the affected and unaffected arm, analysis via Mann–Whitney U and for non-parametrical comparisons of >3 groups (varus, valgus, physiologic) Kruskal–Wallis analysis was conducted for significance. For paired samples, our data were non-parametric, which suggested the use of the Wilcoxon test. Spearman’s Rho correlation was calculated for non-parametric data (relative strength and all PROMS reported *p*-values are two-tailed, with a well-established alpha level of <0.05 considered statistically significant. Unless otherwise stated, descriptive results are demonstrated as mean ± standard deviation (SD) and range (r).

## 3. Results

### 3.1. Patients’ Characteristics

A total of 25 patients, 19 female and 6 males, were enrolled with a mean age of 73.2 ± 10.5 years and a mean follow-up time of 4.5 ± 1.9 years.

Table 1 displays demographic data of the patient collective, including fracture morphology and reduction quality, as well as complications and PROMs.

The patient collective had various medical preconditions. In total, fourteen patients had previously been diagnosed with osteoporosis, six patients had cardiac preconditions, three with atrial fibrillation and 1 with a history of myocardial infarction. Three patients had renal disease and pulmonary conditions. Two patients had an ongoing malignancy. Two patients had neurologic conditions. There was one patient with a history of stroke and diabetes mellitus.

Postoperative physiotherapy was prescribed to every patient for outpatient rehabilitation. In total, 24 of 25 patients attended physiotherapy. Thereof, 10 patients received ≤12 h, 10 patients up to 100 h and 4 patients even more hours of physiotherapeutic training. Additionally, 23 patients did additional exercises themselves, with 76% (*n* = 19) including strengthening exercises for the shoulder girdle. No significant relationship between duration of physiotherapy (≤12 h, <100 h, >100 h) and shoulder function was observed (flexion ROM: *p* = 0.481; abduction ROM: *p* = 0.798; AF_rel_: *p* = 0.541, FF_rel_: *p* = 0.865). On average, mean physical activity for one week did not differ at the point of follow-up (3:37 h pre-injury vs. 3:27 h at follow-up) (*p* = 0.121).

Table 2 reports the relative strength values in isometric force for AF_rel_, FF_rel_ and HG_rel_.

Performing an independent-samples Mann–Whitney U test hypothesising existing differences of relative strength measures between sexes did not show significant differences of relative strength in all three measures (AF_rel_: *p* = 0.366, FF_rel_: *p* = 0.828, HG_rel_: *p* = 0.328)

Significant lower force outputs of the affected arm were found compared to the unaffected arm concerning flexion and abduction forces. Relative differences in flexion force and abduction force (*p* = 0.72) as well as absolute values (*p* = 0.21) did not differ significantly, indicating a likewise reduction of both shoulder forces (abduction and flexion) after injury.

Additionally, no difference in strength measurements was obtained with regards to injury of the dominant or non-dominant side; hereby, AF_rel_ and FF_rel_ as well as HG_rel_ were identical in both groups, after injury of the dominant or non-dominant side (*p* = 0.194, *p* = 0.124, *p* = 0.374).

### 3.2. Influence of Head–Shaft Angle on Strength Outcome

**Figure 3:**
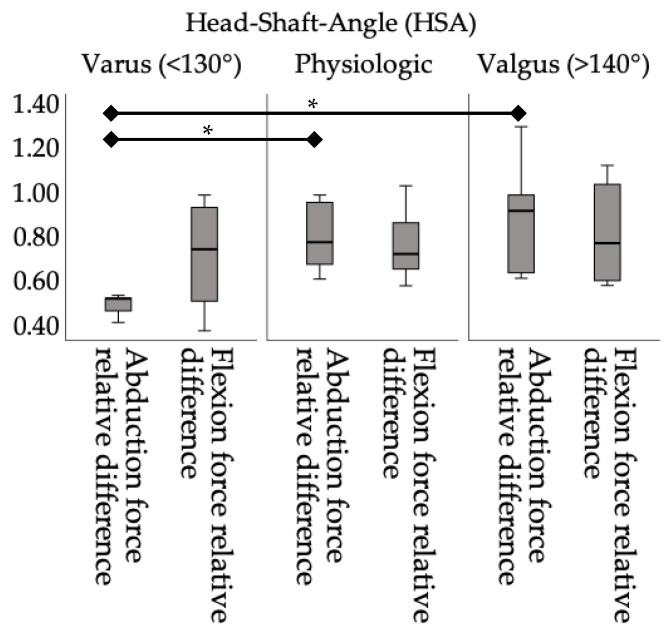
Relative strength differences according to the head–shaft angle group. * = *p* < 0.05.

When categorizing the data concerning HSA (varus, physiologic and varus), the subclassification significantly influences force output during isometric strength testing. Patients in the varus group showed only 50.41 ± 5.13% of isometric abduction force compared to their unaffected arm, compared to 81.65 ± 28.10% and 90.65 ± 25.18% in the physiologic and valgus groups, respectively (*p* = 0.468). This significant decrease in abduction force for varus-angulated humeral heads is a meaningful finding (*p* = 0.014) (see Figure 3). Flexion force appeared unaffected by head-angulation (*p* = 0.522).

The valgus group had the lowest restrictions in ROM (abduction: −23.00 ± 9.75° for varus, −22.14 ± 22.59° for physiologic, −10 ± 8.94° for valgus; flexion: −17.00 ± 10.95° for varus, −19.29 ± 15.67° for physiologic, −11.67 ± 15.71° for valgus). However, these differences were not significant (abduction: *p* = 0.751; flexion: *p* = 0.939).

### 3.3. Hand Grip Strength

Statistical analysis yielded a significant negative difference between the hand grip strength of the affected arm to the unaffected arm (*p* = 0.009). However, since most patients have injured their non-dominant, presumably weaker arm. Further hypothesis testing yielded no statistical significance correcting for this confounder. In dominant-side injuries, the mean hand grip force of the affected and unaffected arm was 26.88 kg and 26.5 kg, respectively (*p* = 0.833). Contrarily, for non-dominant side injuries, the affected arm showed statistically significant negative differences of the affected arm (25.01 kg and 28.12 kg, *p* = 0.001).

### 3.4. Influence of Fragment Count on Strength Outcome

Analysing two-, three-, or four-part fractures yielded different relative strength outputs in abduction force, although missing statistical significance: In two-part fractures of the surgical neck, AF_rel_ is 82.17%, which further decreases to three-part fractures (AF_rel_: 76.22%) and two four-part fractures (AF_rel_: 75.53%) (*p* = 0.939). For FF_rel_, this trend is not consistent (two-part FFrel: 76.67%; three-part FF_rel_: 75.00%; four-part FF_rel_: 82.63%) (*p* = 0.878). A statistically significant decrease of CMS_rel_ from 96.1% in two-part to 89.4% in three-part to 78.3% in four-part PHFs (*p* = 0.01) may be attributable to decreased general function rather than significant ramifications of strength.

### 3.5. Relation of PROMs and Shoulder Strength

A comparison of independent variables yielded significant differences of FF_rel_ and patient-reported satisfaction with the surgery (*p* = 0.046). For AF_rel,_ no significant difference was found (*p* = 0.087). To illustrate, patients who rated the surgery with 1 “very good” (*n* = 11) exhibited on average an AF_rel_ of 90.83%, whereas patients who rated a grade 2 “good” (*n* = 12) produced only an AF_rel_ of 68.46%. For FF_rel_, the same trend was observed, where patients who rated “very good” and “good” achieved a FF_rel_ of 86.98% and 69.65%, respectively. Only three patients rated the result as “satisfactory” and demonstrated an AF_rel_ of 59.17% and a FF_rel_ of 57.67%. Regarding ASES, a significant positive correlation with AF_rel_ of the affected arm (ρ = 0.48; *p* = 0.02) and hand grip force of the affected arm (ρ = 0.41, *p* = 0.04) was demonstrated. In the same sense, a missing correlation of the unaffected arm with ASES reaffirms these findings.

### 3.6. Time to Follow-Up and Shoulder Function

Although displaying heterogenic times to follow up ranging from 1.9 years to 7.6 years, thus variance in possible rehabilitation improvements, shoulder function did not differ accordingly. By Kruskal–Wallis analysis for independent samples with defined groups by years to follow up no statistically significant difference was examined in the tested parameters (flexion ROM: *p* = 0.118, abduction ROM: *p* = 0.439; AF_rel_: *p* = 0.741; FF_rel_: *p* = 0.809). Therefore, a sufficient and statistically equal level of rehabilitation between the patients was assumed.

## 4. Discussion

Firstly, our investigation revealed a significant reduction of strength in the two most important shoulder movements, i.e., flexion and abduction, following years after surgery. Standardized strength measurements, i.e., with a handheld static dynamometer, seem valuable for the investigation of shoulder joint function after surgery. Secondly, patients with varus-angulated head–shaft angles have produced significantly less abduction force compared to physiologic or even valgus angulation, possibly due to leverage-arm deteriorations and early subacromial impingement. Thirdly, we have identified a significant reflection of the strength parameters in patients’ subjective satisfaction with the surgery and PROMs, which undermines the clinical relevance of strength testing.

In our measurements, force in both degrees of freedom has shown to be reduced by about a quarter compared to the healthy, unaffected arm. To our knowledge, this is the first publication of raw isometric strength data after intramedullary nailing of proximal humerus fractures, and it is a challenge to contextualize these values. Despite the vast number of studies and meta-analyses comparing IMN with locking plates or conservative treatment for proximal humerus fractures regarding reduction quality, complications or PROMs, there is no comprehensive juxtaposition of long-term strength outcomes [8,21]. Standing alone, the randomized controlled trial by Zhu et al. (2011) with 51 subjects concluded a significantly higher relative abduction strength in the locking plate group compared to IMN (77.4% compared to 64%) one year postoperatively (*p* = 0.032) [10]. In their three-year postoperative follow-up, there were no differences (*p* = 0.106), contradicting our results. Unfortunately, the authors did not specify the methodology of the measurements and whether isometric or isokinetic data were involved.

Additionally, Verdano et al. (2014) have analysed isokinetic flexion–extension and internal–external rotation strength in patients with PHFs treated with open reduction and internal fixation (ORIF), i.e., locking plates and reported no statistically significant difference in flexion–extension peak torque but a notable trend [13]. Meanwhile, their CMS values differed between shoulders: their explanation infers patient’s subjective perception of worse function or residual pain creates these differences, which is a statement our data cannot reinforce. Another possible explanation for the discrepancies between CMS and Verdano’s strength values could be the absence of an evaluation of abduction strength, which appears to play a critical role following proximal humeral fractures and is evaluated in the CMS, potentially accounting for a maximum of 25% of points.

Further, although our strength measurements show significant differences between sides, they may still be underestimated due to the exclusion of patients with presumably below-average shoulder function: patients not reaching testing positions and patients treated with RSA as revision surgery following IMN.

The substantial influence of the head–shaft angle on isometric abduction force stands as the pivotal discovery. To our knowledge, up until now there exist no studies analysing the influence of the head–shaft angle on clinical shoulder strength.

A possible reason for presumably worse abduction force in varus-angulated humeral heads could be a superomedial shift of the greater tuberosity and, therefore, a reduced capacity of the pre-shortened supraspinatus muscle to produce force. Although it is commonly believed that its contribution to shoulder abduction ceases after the first 20 to 40°, there are studies showing a contribution during abduction in the frontal plane far beyond that [22]. Also, this finding was not observed to the same degree for flexion force. Reasoning, this movement in the sagittal plane is mainly generated by the anterior fibres of the deltoid and pectoralis major muscle, which are anatomically not impacted by HSA differences, which are constituted in the frontal plane.

Another factor can be the increased risk of early subacromial impingement during abduction due to the varus-angulated head, possibly impeding maximum strength output in the pain-provoking position where the subacromial space is most narrow. It is important to add that these observations are unlikely attributable to the training effect or any methodological errors since flexion force was tested before the abduction force. In the future, there may be room for appropriate biomechanical studies, if our findings can be reproduced.

Adjunctively, we decided to report the well-established dynamometer grip force measurements as a prognostic geriatric indicator for frailty and overall body strength for patients who obtained PHFs. Our results illustrate significantly reduced relative hand grip forces on the affected arm even years after the injury (27.03 kgs ± 12.16 -> 24.60 kgs ± 13.14), which may increase the patient’s geriatric decline. Additionally, the effect of PHFs on hand grip force has not been described in previous literature, and there is a known link of grip strength to frailty and sarcopenia, two common geriatric syndromes [23].

Interestingly, we have illustrated that patients with small abduction and flexion force differences between the injured and healthy arms report consistently better overall satisfaction. Similarly, ASES, which is a validated PROMs measure for shoulder fractures, correlates with shoulder strength, indicating clear relevance of the variable “strength” on patient-reported outcomes. Following these investigations, a naturally arising question would be whether these measurable strength reductions have a practical significance in the patient’s life and whether the constraint is seriously contributing to premature loss of independence in the elderly. Notably, patient satisfaction with the procedure was closely tied to strength levels, as evidenced by cohorts reporting lower satisfaction when FF_rel_ and AF_rel_ were more diminished.

Another breaking point for outcome research is the consideration of postsurgical rehabilitation and patient’s lifestyle. All except one patient included in this study have completed the minimum required 12 h of physiotherapy, while many have received considerably more. No difference in ROM or strength outcomes was observed, regarding the amount of physiotherapeutic training. Here, one must acknowledge the typical “chicken or the egg” causality dilemma, where more physiotherapy following prolonged functional progression might have benefitted the patient in the end.

The present results of this pilot study should be evaluated considering specific limitations. First, this study included a small number of subjects, which is a considerable limitation, especially regarding the analysis of subgroups. For future endeavours, it would be of great interest to challenge our results in a larger patient collective. Secondly, considering the mid- to long-term follow-up time, subjects were not standardized for their different rehabilitation regime or physiotherapy, which may be a confounding variable. Finally, presuming strength reductions following surgery is based on the assumption that the contralateral arm at the point of follow-up depicts the identical (theoretical) strength of the affected arm if the injury (PHF) did not occur. Since most patients fractured their non-dominant arm, the pre-injury shoulder strength may have been lower than the contralateral shoulder beforehand.

## 5. Conclusions

Proximal humerus fractures cause significant strength loss in the affected shoulder, particularly in the elderly, regardless of fracture complexity or anatomical reduction. Our findings indicate that surgical reduction leading to a varus head–shaft angle should be avoided due to the associated reduction in abduction force. Isometric strength measurements are valuable clinical indicators that should be included in further research with more participants and clinical shoulder scoring systems to enhance the accuracy of evaluations. Incorporating these measurements provides comprehensive insights into muscle function, improving the validity of research findings and patient care in shoulder-related conditions.

## Figures and Tables

**Table 1:** Summary of descriptive statistics for metric and nominal data. Metric data are reported as mean ± standard deviation and range, nominal data as total number *n* and associated percentages (%).

	Total	Female	Male
Gender	*n* = 25	*n* = 19	*n* = 6
Age	73.2 ± 10.5 (SD) r: 55–88	74.63 ± 10.84 (SD) r: 55–88	68.50 ± 8.55 (SD) r: 56–81
≥65 years	18 (72.0%)	14 (73.69%)	4 (66.7%)
<65 years	7 (28.0%)	5 (26.31%)	2 (33.3%)
Follow up (years)	4.50 ± 1.88 (SD) r: 1.9–7.6	4.45 ± 1.75 (SD) r: 2.0–7.6	4.68 ± 2.42 (SD) r: 1.9–7.6
Injured side			
right	*n* = 9 (36%)	*n* = 6	*n* = 3
left	*n* = 16 (64%)	*n* = 13	*n* = 3
dominant	*n* = 8 (32%)	*n* = 7	*n* = 1
non-dominant	*n* = 17 (68%)	*n* = 12	*n* = 5
Fracture parts			
two-part	*n* = 6 (24%)	*n* = 2	*n* = 4
three-part	*n* = 16 (64%)	*n* = 14	*n* = 2
four-part	*n* = 3 (12%)	*n* = 3	*n* = 0
Head–shaft angle postoperatively	135.8 ± 7.9° (SD); r: 124.8–151.8°		
Varus < 130°	*n* = 5 (20%)	*n* = 4	*n* = 1
physiologic	*n* = 14 (56%)	*n* = 9	*n* = 5
valgus > 140°	*n* = 6 (24%)	*n* = 6	*n* = 0
Complications according to Clavien–Dindo			
I or II i.e., “minor”	*n* = 1 (4%)	*n* = 1	*n* = 0
III i.e., “major”	*n* = 5 (20%)	*n* = 4	*n* = 1
Implant failure	*n* = 4 (16%)	*n* = 3	*n* = 1
PROMs			
ASES Score	85.40 ± 16.6 (SD) r: 31.7–100	81.84 ± 17.55 (SD) r: 31.66–10.00	96.67 ± 5.35 (SD) r: 88.32–100.00
CMS affected arm	69.72 ± 13.13 (SD) r: 39.0–92.0	68.05 ± 10.60 (SD) r: 40.00–78.00	75.00 ± 19.46 (SD) r: 39.00–92.00
CMS unaffected arm	77.68 ± 11.10 (SD) r: 40.0–98.0	76.53 ± 5.73 (SD) r: 58.00–84.00	81.33 ± 21.27 (SD) r: 40.00–98.00
CMS_rel_	89.67 ± 10% (SD) r: 57.14–101.3%	88.75 ± 11.18% r: 57.14–101.30%	92.56 ± 4.15% r: 85.19–97.50%

**Table 2:** Absolute and relative force measurements of the affected and unaffected arm as well as Constant–Murley scores (CMS).

	Affected Arm Force	Unaffected Arm Force	Relative Force Difference	*p*-Value
Absolute isometric abduction force (N ± SD)	2.90 ± 1.95 kg	3.86 ± 2.54 kg		*p* < 0.001
Relative isometric abduction force (range)	0.64–8.07 kg	1.36–10.70 kg	−24.47% (AFrel)	
Absolute isometric flexion force (N ± SD)	3.13 ± 1.81 kg	4.21 ± 2,27 kg		*p* < 0.001
Absolute isometric flexion force (range)	1.09–8.26 kg	1.63–10.7 kg	−25.30% (FFrel)	
Absolute isometric hand grip force (N ± SD)	25.64 ± 13.5 kg	27.6 ± 12.4 kg		*p* = 0.003
Relative isometric hand grip force (range)	12.0–67.0 kg	13.0–60.0 kg	−8.30% (HGrel)	
CMS relative ± SD	69.72 ± 13.1	77.68 ± 11.1		*p* < 0.001
Range	39.0–92.0	40.0–98.0	−10.25%	

## Data Availability

Data are contained within the article.
